# Association of the protein tyrosine phosphatase non-receptor 22 polymorphism (*PTPN22*) with endometriosis: a meta-analysis

**DOI:** 10.1590/S1679-45082017RW3827

**Published:** 2017

**Authors:** Noel Pabalan, Hamdi Jarjanazi, Denise Maria Christofolini, Bianca Bianco, Caio Parente Barbosa

**Affiliations:** 1 Cebu Doctors’ University, Cebu, CE, Canada.; 2 Ontario Ministry of the Environment and Climate Change, Ontario, ON, Canada.; 3 Faculdade de Medicina do ABC, Santo André, SP, Brazil.

**Keywords:** Protein tyrosine phosphatases, non-receptor, Polymorphism, genetic, Endometriosis

## Abstract

**Objective:**

To evaluate *PTPN22* C1858T polymorphism and the risk of endometriosis.

**Methods:**

A meta-analysis of 10 published case-control studies (from four articles), with a total sample of 971 cases and 1,181 controls, was performed. We estimated risk (odds ratio and 95% confidence intervals) of endometriosis associations with the C1858T polymorphism.

**Results:**

A significant increased risk in all genetic models of the variant T allele with endometriosis (odds ratio: 3.14-5.55; p<0.00001-0.002) was found. The analysis without the study whose controls deviated from the Hardy-Weinberg equilibrium exacerbated these effects in the homozygous and recessive models (odds ratio: 7.19-9.45; p<0.00001-0.0002). In the Italian subgroup, a significant risk association was found in the homozygous and recessive models (odds ratio: 8.72-11.12; p=0.002).

**Conclusion:**

The associations observed between *PTPN22* (C1858T) and the risk of endometriosis suggest this polymorphism might be a useful susceptibility marker for this disease.

## RESUMO

**Objetivo:**

Avaliar o polimorfismo *PTPN22* C1858T e o risco de endometriose.

**Métodos:**

Foi realizada uma metanálise de 10 estudos caso-controle publicados (a partir de quatro artigos), com uma amostra total de 971 casos e 1.181 controles. O risco da associação da endometriose com o polimorfismo C1858T foi estimado em razão de chance e intervalo de confiança de 95%.

**Resultados:**

Observou-se um aumento de risco significativo em todos os modelos genéticos com o alelo variante T e a endometriose (razão de chance: 3,14-5,55; p<0,00001-0,002). A análise sem incluir o estudo, em que os controles não estavam em equilíbrio de Hardy-Weinberg, mostrou aumento significativo nos modelos homozigotos e recessivos (razão de chance: 7,19-9,45; p<0,00001-0,0002). No subgrupo italiano, uma associação significativa foi encontrada considerando os modelos homozigoto e recessivo (razão de chance: 8,72-11,12; p=0,002).

**Conclusão:**

As associações observadas entre *PTPN22* (C1858T) e o risco de endometriose sugerem que este polimorfismo pode ser um marcador de suscetibilidade para a endometriose.

## INTRODUCTION

Endometriosis is a condition in which a tissue that is histologically similar to the endometrium, with glands and/or stroma, grows outside the uterine cavity.^1^ It is a chronic inflammatory disease and one of the most common benign gynecological disorders. It presents multisystem involvement affecting several organs, most commonly in the peritoneum and pelvis, especially the ovaries, and less often in the recto-vaginal septum.^2^ This results in pelvic pain, dysmenorrhea, and infertility.^3^

Numerous hypotheses have already been put forward to explain the presence of ectopic endometrial tissue and stroma. However, none of them could explain all implantation sites and symptoms, leading researchers to search for new theories which alone or together with the hypotheses already proposed could better explain the etiology of endometriosis.

Exposure to estrogen is one of the major endocrine risk factors for endometriosis. In contrast, progesterone is somehow protective against the development of endometriosis, for disrupting the production of local differentiation factors necessary for the regulation of the expression of responsible genes, promoting the ability of refluxed menstrual endometrial fragments to invade the peritoneal surface, interfere in vessels, and establish endometriosis. Because of the powerful anti-inflammatory effect of progesterone, reduced sensitivity to this steroid could contribute to the autoimmune nature of endometriosis.^4,5^

Although the etiology of autoimmune diseases is unknown, they are characterized by genetic and environmental factors in their development. Just as in autoimmune diseases, in endometriosis similar immunologic alterations occur, such as an increase in the number and cytotoxicity of macrophages, polyclonal increase in the activity of B lymphocytes, abnormalities in the functions and concentrations of B- and T-lymphocytes, and reduction in the number or the activity of natural killer cells. Furthermore, the presence of specific antiendometrial and antiovary antibodies has been found both in endometriosis and infertility.^4,6,7^In this context, hypotheses addressing immunological predispositions as well as genetic factors have been considered,^4,8^ and polymorphisms in genes associated with autoimmune diseases have emerged as possible candidates for endometriosis development.^4^

Lyp is a protein tyrosine phosphatase encoded by non-receptor 22 (*PTPN22*) gene, located on 1p13.3-13.1, and it is involved in the regulation of T-cell receptor signaling.^9^The *PTPN22* gene shows a missense single-nucleotide polymorphism at nucleotide 1858 (C>T), which causes a substitution of an arginine at codon 620 (CGG) for a tryptophan (TGG) (W620 variant) associated with autoimmune disorders.^10^ The variant does not bind kinases well and appears to encode a gain-of-function enzyme, which has been suggested to increase the inhibition of T-cell-receptor signaling, affecting thymic deletion of autoreactive T-cells or the development or function of peripheral regulatory T-cells.^11,12^

The *PTPN22* polymorphism was reported to be associated with altered risk of endometriosis, although with conflicting results, prompting us to conduct a meta-analysis to evaluate this association.

## OBJECTIVE

To evaluate the association between *PTPN22* C1858T polymorphism and the risk of endometriosis.

## METHODS

### Selection of studies

By means of the terms “*PTPN22* polymorphism” and “endometriosis”, we searched MEDLINE using PubMed for association studies as of April 27, 2014. Studies were eligible if they had genotypic data with a case-control design. The search yielded seven citations, two of which were excluded as they were review papers and not about endometriosis. Abstracts of the remaining five citations were read, and one was excluded because it was not about *PTPN22*. Full-text articles of the remaining four were extracted, read, and determined to be included in the meta-analysis.^13-16^ Ammendola et al.^13^ and Gloria-Bottini et al.^16^ provided two and six sets of genotypic datasets, respectively, and were considered two and six studies, respectively. Added to the two articles^14,15^ with singular datasets, the final number of studies included in the meta-analysis was ten.

### Data extraction

Two investigators independently extracted data and reached consensus on all items. The following information was obtained from each publication: first author’s name, year of publishing, country of origin, dominant ancestry of the study populations, status of controls, matching criteria, sample source, genotyping approach, genotype data, number of cases and controls. We also calculated frequencies of the variant allele, and deviations of the controls from the Hardy-Weinberg equilibrium (HWE).

### Quality assessment of the studies

The Newcastle-Ottawa Score (NOS) quality assessment scale^17^ was used to assess the methodological quality of the studies included. These studies were judged based on three broad perspectives: selection, comparability, and exposure (case-control studies) or outcome (cohort studies), by a ‘star’ rating system with a score ranging from zero star (worst) to nine stars (best). A score of seven stars or greater indicated that one study was of high quality.

### Meta-analysis

Data were analyzed using Review Manager 5.1 (Copenhagen: The Nordic Cochrane Centre, The Cochrane Collaboration, 2011). We estimated the odds ratio (OR) of association with the variant TT genotype compared with the wild-type CC genotype. To evaluate importance of the heterozygous genotype, dominant and recessive genetic models were also applied. Thus, we examined contrast of TT *versus* TC + CC genotypes as well as the TT + TC *versus* CC genotypes. These contrasts correspond to recessive and dominant effects of the T allele. To compare effects on the same baseline, we used raw data for genotype frequencies to calculate study-specific estimates of the OR. Pooled ORs were obtained using either the fixed^18^ (in the absence of heterogeneity) or random^19^ (in its presence) effects models. Heterogeneity between studies was addressed in a number of ways. First, it was estimated using the χ^2^ based Q test.^20^ Recognizing the low power of this test,^21^ significance threshold was set at p=0.10. Second, it was explored using subgroup analysis^20^ with population as variable. And third, it was quantified with the I^2^statistic, which measures the degree of inconsistency among studies.^21^ Sensitivity analysis, which involved omitting one study at a time and recalculating the pooled OR, was also used to test for robustness of the summary effects. Significance was set at a p value of ≤0.05 throughout, except in heterogeneity estimation.

### Publication bias

Ammendola et al.,^13^ had zero homozygous and recessive datasets in cases and controls, which rendered them non-estimable, hence the total number of studies was nine for these genetic models. When the number of studies is lower than ten,^22^ qualitative and quantitative tests for publication bias become less sensitive, obviating investigation of publication bias. Non-zero data in the dominant and co-dominant models placed the overall total number of studies at 10, warranting test of publication bias in these models. In this case we used the regression asymmetry test by Egger et al.,^23^ as well as the diagnosis by Begg et al., (nonparametric σ correlation coefficient).^24^ For both tests, we used the web-based software, WINPEPI (PEPI for Windows).^25^

## RESULTS

### Included studies

The epidemiological and clinical characteristics of the included articles are outlined in table 1. The NOS results showed that three out of four articles scored seven, and the mean score was x̅=6.75±0.50. These two features indicate that the methodological quality of the articles was medium to high. Table 2 summarizes the quantitative features of the ten genotyping studies (443 cases/1,181 controls) in the meta-analysis that examined associations of the *PTPN22* (C1858T) polymorphism with endometriosis. Eight studies from two articles^13,16^ had Italian subjects (132 cases/528 controls), while one study had Brazilian subjects^15^ and another had Polish participants,^14^ with 140 cases/180 controls and 171 cases/310 controls, respectively.

**Table 1:** Characteristics of the included articles that examined protein tyrosine phosphatase non-receptor 22 (C1858T) associations with endometriosis

First author	PY	Country	Number of studies	Ethnic group	Study design	Diagnosis of endometriosis	Controls	Matching	NOS
Ammendola et al.^(13)^	2008	Italy	2	Caucasian	PB	Laparoscopic intervention	163 males and 69 females, both healthy	Not matched	7
Płoski et al.^(14)^	2009	Poland	1	Caucasian	PB	Laparoscopic and histopathological examinations	310 anonymous unrelated adults representative of the population of Central Poland. No information on affliction status regarding endometriosis	Matched by ethnicity	7
Gomes et al.^(15)^	2010	Brazil	1	Multi-ethnic	PB	Laparoscopic and histopathological examinations	180 fertile non-menopausal women without history of endometriosis and/or autoimmune diseases who underwent tubal ligation	Not matched	6
Gloria-Bottini et al.^(16)^	2013	Italy	6	Caucasian	PB	Laparoscopic intervention; histologically confirmed	359 healthy blood donors	Not matched	7

PY: publication year; PB: population-based; NOS: Newcastle-Ottawa Score.

**Table 2:** Frequency of genotypes and other characteristics of the included studies regarding association of protein tyrosine phosphatase non-receptor 22 (C1858T) with endometriosis

First author	Study identifier*	Genotype frequencies						Minor allele frequencies	Hardy-Weinberg equilibrium

		Case			Control				
	
		CC	CT	TT	CC	CT	TT		
Ammendola et al. ^(13)^	Female controls ♀	110	22	0	67	1	1	0.02	<0.0001
Ammendola et al. ^(13)^	Male controls ♂	110	22	0	151	12	0	0.04	0.63
Płoski et al.^(14)^	-	129	38	4	238	68	4	0.12	0.73
Gomes et al.^(15)^	-	95	42	3	149	29	2	0.09	0.66
Gloria-Bottini et al.^(16)^	ACP1 C allele	13	17	5	319	39	1	0.06	0.87
Gloria-Bottini et al.^(16)^	ACP1 other genotypes	75	21	1	319	39	1	0.06	0.87
Gloria-Bottini et al.^(16)^	P53 codon Pro allele	44	24	4	319	39	1	0.06	0.87
Gloria-Bottini et al.^(16)^	P53 codon Arg/Arg genotype	48	11	1	319	39	1	0.06	0.87
Gloria-Bottini et al.^(16)^	Duration >6 months	25	19	4	319	39	1	0.06	0.87
Gloria-Bottini et al.^(16)^	Duration <6 months	70	13	1	319	39	1	0.06	0.87

* Independent genotypic data were presented for each study identifier enabling calculation of pooled odds ratio.

Control frequencies of the variant allele in the Italian studies ranged from 0.02 to 0.06, while Brazilian and Polish populations were 0.09 and 0.12, respectively. One study^13^ had controls whose frequencies deviated from the HWE, leaving nine studies from three articles^11-13^ in HWE (311 cases/1,012 controls).

### Overall and subgroup analysis

**Figure 1:**
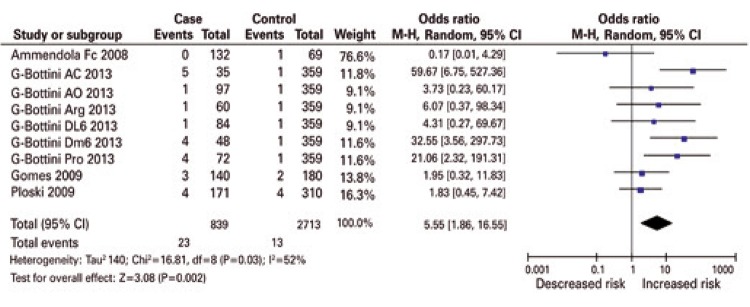
Overall recessive summary effect of the protein tyrosine phosphatase non-receptor 22 (C1858T) polymorphism with endometriosis

**Figure 2:**
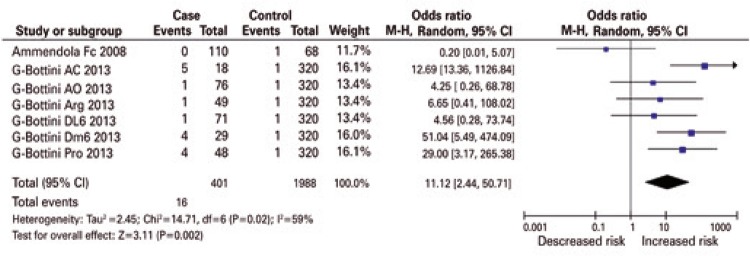
Recessive summary effect of the protein tyrosine phosphatase non-receptor 22 (C1858T) polymorphism with endometriosis without the study whose controls deviated from the Hardy-Weinberg equilibrium

**Figure 3:**
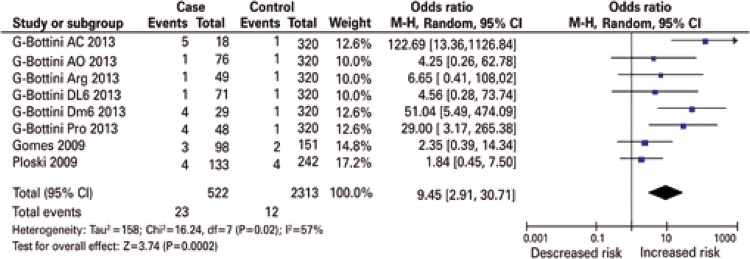
Summary effects of protein tyrosine phosphatase non-receptor 22 on endometriosis in the homozygous model

Figure 1 shows the significantly increased (p<0.0001-0.002) overall risk effects (OR: 3.14-5.55, 95% confidence interval 95%CI: 1.86-16.55) in all genetic models. The forest plots show that virtually all study-specific ORs lie within the area of increased risk in all genetic models (Figures 1 to 3). No publication bias was observed in the dominant and co-dominant models (Table 3). By confining the studies to those in HWE, the overall findings changed in two manners: (i) effects were exacerbated up to OR: 9.45 (p=0.0002) in the homozygous model (Table 4 and Figure 2) and (ii) received even wider CIs (95%CI: 2.91-30.71).

**Table 3:** Summary of pooled effects

	Number of studies	Homozygous			Recessive			Number of studies	Dominant			Co-dominant		
			
		OR (95%CI) p value	P_het_	I^2^	OR (95%CI) p value	P_het_	I^2^		OR (95%CI) p value	P_het_	I^2^	OR (95%CI) p value	P_het_	I^2^
Overall	9	5.55 (1.86-16.55) 0.002	0.03	52	3.23 (1.86-5.59) <0.0001	<0.0001	85	10	3.14 (1.91-5.17) <0.0001	<0.0001	83	3.14 (1.93-5.10) <0.0001	<0.0001	86
HWE only	8	9.45 (2.91-30.71) 0.0002	0.02	57	7.19 (2.61-19.82) <0.0001	0.09	43	9	2.99 (1.78-5.02) <0.0001	<0.0001	85	3.08 (1.84-5.14) <0.0001	<0.0001	88
Italian studies only	7	11.12 (2.44-50.71) 0.002	0.02	59	8.72 (2.27-33.53) 0.002	0.07	49	8	3.83 (2.28-6.45) <0.0001	<0.0001	76	3.86 (2.40-6.21) <0.0001	<0.0001	78
Brazil*	1	2.35 (0.39-14.34)	-	-	1.95 (0.32-11.83)	-	-	1	2.28 (1.35-3.85)	-	-	2.05 (1.28-3.29)	-	-
Poland*	1	1.84 (0.45–7.50)	-	-	1.83 (0.45-7.42)	-	-	1	1.08 (0.70-1.67)	-	-	1.11 (0.75-1.65)	-	-

* Odds ratio and 95% confidence interval are study-specific values.

OR: odds ratio; 95%CI: 95% confidence interval; P_het_: p value for heterogeneity; I^2^ are expressed in %.

**Table 4:** Tests for publication bias

	Number of studies	Egger Regression		Begg Mazumdar	
	
		Intercept	p value	Kendall’s t	p value
Dominant	10	4.85	0.11	0.38	0.13
Co-dominant	10	4.07	0.26	0.24	0.33

* Independent genotypic data were presented for each study identifier enabling calculation of pooled odds ratio.

Table 4 shows the summarized effects in the Italian subgroup and compares them with the study-specific ORs of the Brazilian^12^ and Polish^14^ populations. The significant Italian effects increased up to OR: 11.12 in the homozygous model, accompanied by wide confidence intervals (95%CI: 2.44-50.71) (Figure 3). Figures 2 and 3 show that the HWE and Italian pooled effects may have been attributed to the ACP1 genotype C allele study, by Ammendola et al.,^13^ with their study-specific OR of 122.69 and 95%CI: 13.36-1126.84.

In contrast to the Italian pooled effects, the study-specific ORs were modulated in the Brazilian (OR: 1.95-2.35) and Polish (OR: 1.08-1.84) populations. Sensitivity treatment did not materially alter the overall and HWE results as the Italian findings did, thus indicating robustness of the summary effects (data not shown). All 12 pooled ORs were heterogeneous, high in the dominant and co-dominant models (P_heterogeneity_<0.00001, I^2^=76-88%), less in the homozygous and recessive models (P_heterogeneity_=0.02–0.09, I^2^=43–59%).

## DISCUSSION

### Overall and subgroup effects

With a sample of over 1,624 for the *PTPN22* (C1858T) polymorphism, our meta-analysis showed overall increased risk associations of up to 5.6-fold in endometriosis, significant in all genetic models. The HWE analysis did not materially alter the overall findings, other than exacerbate susceptibility up to 9.5-fold in the homozygous model. Interpreting such an increase should, however, be treated with caution given the unusually wide CI that accompanied the pooled effects. Wide CI margins tend to heighten uncertainty, hence, less confidence in the interpretation of results.

Because there was only one of each Brazilian and Polish studies and several Italian studies, we compared these single-ethnic populations with the pooled Italian effects. By and large, the Italian effects were significant up to 11-fold in the homozygous model with the widest CIs in the entire body of results and heterogeneous. By comparison, increased risk effects in the Brazilian population^12^ was up to 2.4-fold, and a comparably modest 1.8-fold in the Polish population.^14^

These differences among the three populations may be associated with the following factors: (i) the minor allele frequencies between these three populations differed (Italian: up to 0.06; Brazilian: 0.09, and Polish: 0.12), (ii) controls in Polish study^14^ were matched to cases compared to none in the Italian subjects. Thus, it may be one or a combination of genetic (allele frequencies) and/or epidemiological (matching of subjects) features that rendered differences in summary effects among the three populations.

However, the following features of the studies account for the advantages of the non-Italian subgroup: (i) the homozygous and recessive effects in this population were obtained in zero heterogeneity; (ii) although composed of only two studies (of the total 10), their combined sample sizes (n=801) accounts for more than one-third (37.2%) of the total 2,152; (iii) findings in this population had the narrowest CIs, boosting precision of the findings. Given these features of the non-Italian studies, it may be that these values may be closer to the true values of association. More studies are needed to confirm our findings. Nevertheless, the observed associations between *PTPN22* and risk of endometriosis suggest this polymorphism might be a useful susceptibility marker for this disease.

### Functional associations of protein tyrosine phosphatase non-receptor 22 (C1858T) with endometriosis

The *PTPN22* gene encodes the human lymphoid tyrosine phosphatase, an enzyme with restricted expression in hematopoietic cells. Lymphoid tyrosine phosphatase is a critical regulator of signaling through the T-cell receptor, and in T-cells, it forms a complex with the kinase Csk.^8^ The autoimmune-associated *PTPN22* C1858T variant does not bind kinases well, and appears to encode a gain-of-function enzyme.^12,26^ The mechanism of action of *PTPN22* in autoimmunity remains unclear. However, increased inhibition of T-cell receptor signaling caused by the *PTPN22* C1858T polymorphism could predispose towards autoimmunity, either by affecting thymic deletion of autoreactive T-cells or by affecting development or function of peripheral regulatory T-cells.^27^ Indeed, recently, *PTPN22* was found to be among the gene targets of FoxP3 in CD4+CD25+ regulatory T-cells.^28^

In the presence of endometriosis, the *PTPN22* polymorphism may cooperate with clinical and genetic factors to influence the course of disease and immune reactions. These cooperative interactions could result in a statistical association between *PTPN22* and endometriosis. Further investigations are needed to clarify the possible role of *PTPN22* and other polymorphic systems in the clinical course of endometriosis. In subjects with endometriosis, *PTPN22* may contribute to the development of autoimmune phenomena in the presence of peculiar circumstances.^13^ Given the multifactorial nature of endometriosis, the analysis of genetic factors would be proper when considering the synergy with environmental influence along with epistatic interactions.^16^

### Limitations and strengths

Limitations of our study include the following: (i) predominant heterogeneity of the body of results indicating variance of the component studies, which may have been offset by our adjustment for this variance with use of the random-effects model; (ii) deviation of one study^13^ from the HWE, which may have biased summary outputs and pointed to methodological weaknesses, such as biased selection of subjects, genotyping errors, and population stratification.^29^ However, omitting this study followed by reanalysis did not materially alter significance and direction of association, underpinning the stability of our overall findings; (iii) the homozygous and recessive effects were characterized by unusually wide CIs in the overall analysis, which got even wider in the modifier and subgroup analyses and translate to reduced precision of the pooled ORs eliciting less confidence in the findings; (iv) there was no mention of matching in all but one^14^ of the component articles.

Additionally, a possible limitation could be the heterogeneity of the Control Group (v), as healthy men and women,^13^ anonymous healthy adults,^14^ and healthy blood donors.^16^ Only one study^15^had as Control Group fertile and non-menopausal women, who had undergone tubal ligation for family planning reasons, and had no sign of endometriosis in their clinical history. The absence of symptoms in women does not exclude endometriosis, given that 16% of patients with endometriosis are fertile and asymptomatic.^30^

Yet, despite these limitations, the following strengths boost confidence in our findings: (i) all studies were population-based, easing extrapolation of results to the general population; (ii) all tissue sources were blood; (iii) endometriosis diagnoses were all made by laparoscopic intervention and histopathological confirmation; (iv) all studies used a combination of polymerase chain reaction (PCR) and RFLP with enzyme restriction. These five items add to the epidemiological and clinical homogeneity of the studies: consistency of increased risk effects in the entire body of results; sensitivity analysis demonstrated that the entire body of results was robust, supporting the reliability of the findings; and no publication bias was detected, indicating that the dominant and co-dominant body of results may be unbiased.

## CONCLUSION

The findings we report here highlight the utility of modifier analyses which provide a more comprehensive profile of an association of a polymorphism with the disease (endometriosis). Such meta-analytical treatments tend to uncover new insights into factors that retain or alter the stability or robustness of a pooled odds ratio. The synthetic approach to the individual profiles of each included study could be used to form biologically plausible subgroups.

It is conceivable that endometriosis related to any *locus* will be small because gene-gene as well as gene-environment interactions are likely to operate. Additional well-designed studies, based on sample sizes commensurate with detection of small genotypic risks, should allow conclusions that are more definitive as to the association of *PTPN22* (C1858T) polymorphism with endometriosis.
